# Electrochemical Performance of Titania 3D Nanonetwork Electrodes Induced by Pulse Ionization at Varied Pulse Repetitions

**DOI:** 10.3390/nano11051062

**Published:** 2021-04-21

**Authors:** Amirhossein Gholami, Chae-Ho Yim, Amirkianoosh Kiani

**Affiliations:** 1 Silicon Hall, Micro/Nano Manufacturing Facility, Faculty of Engineering and Applied Science, Ontario Tech University, 2000 Simcoe St N, Oshawa, ON L1G 0C5, Canada; amirhossein.gholami@ontariotechu.net; 2 National Research Council Canada, Energy, Mining, and Environment Research Centre, 1200 Montreal Road, Ottawa, ON K1V 0R6, Canada; Chae-Ho.Yim@nrc-cnrc.gc.ca

**Keywords:** nanomaterials, electrodes, titania nanonetwork, pulse ionization, electrochemical

## Abstract

Pulse ionized titania 3D-nanonetworks (T3DN) are emerging materials for fabricating binder-free and carbon-free electrodes for electrochemical energy storage devices. In this article, we investigate the effect of the one of the most important fabrication parameters, pulse frequency, for optimizing supercapacitor efficiency. A series of coin cell batteries with laser-induced electrodes was fabricated; the effect of pulse frequency on oxidation levels and material properties was studied using both experimental and theoretical analysis. Also, detailed electrochemical tests including cyclic voltammetry (CV), charge/discharge, and electrochemical impedance spectroscopy (EIS) were conducted to better understand the effect of pulse frequency on the electrochemical performance of the fabricated devices. The results show that at a frequency of 600 kHz, more T3DN were observed due to the higher temperature and stabler formation of the plasma plume, which resulted in better performance of the fabricated supercapacitors; specific capacitances of samples fabricated at 600 kHz and 1200 kHz were calculated to be 59.85 and 54.39 mF/g at 500 mV/s, respectively.

## 1. Introduction

Lithium-ion batteries (LIBs) and supercapacitors have been finding new applications in electrochemical energy storage (EES) devices in our day-to-day activities as replacements for conventional energy sources and fossil fuel-based internal combustion (IC) [1,2,3]. Recently, both LIBs and supercapacitors have attracted a lot of attention in plug-in/hybrid electric vehicles for their high energy density (kWh kg^−1^) and power density (kW kg^−1^) respectively. In general, supercapacitors demonstrate high power density as well as long cyclic performance. On the other hand, LIBs are known for their high energy density but lower power density and cycle life compared to the supercapacitors [4,5,6,7,8,9].

Electrode materials play an important role in the performance of any energy storage devices including LIBs and supercapacitors [8]. Recently, much research has been focused on replacing commercialized graphite in anodes with alternative materials like TiO_2_. Some of its advantages include being environmentally friendly and chemically stable, and having a low-cost fabrication process. However, due to its low electrical conductivity and ion diffusivity in LIBs, TiO_2_ can limit the storage capacity in EES [10,11,12,13].

Nanostructured titania (TiO_2_) such as anodized titania nanotubes with increased surface area between electrode and electrolyte and shorter diffusion length can overcome the mentioned problem [14,15,16]. Recently, several research results have been reported to improve the electrochemical properties of titania nanotubes by combining it with graphene materials [17], nitrogen doping [18,19], adding metal oxides [20], synthesis of arrays of TiO_2_ nanowire via template sol gel deposition [21,22].

In our recent work [23], we proposed a new approach via pulse ionization for single step and chemical-free fabrication of titania 3D nanonetworks (T3DN) with significantly increased surface area and improved electrochemical properties. T3DN is directly grown from a Ti metal sheet by ultra-short pulse laser ablation. The Ti sheet covered by the growth T3DN is a binder and conducting agent-free active material which minimizes the resistance between the current collector (Ti) and active materials. In the indicated work [23], we only introduced our novel method and provided results for proof of concept. However, the effects of laser parameters including pulse frequency has not been investigated. Both topology and materials properties of the generated nanostructures can be customized by controlling the laser parameters. Thus, more studies needed to be conducted to investigate effects of the fabrication parameters on the electrochemical behaviors of the samples via both Nyquist and Bode plots.

In this research work, we used low and high frequency in laser fabrication process to tune the T3DN electrode electrochemical properties in supercapacitors. We prototyped coin cells using fabricated electrodes. We investigated the EIS results using Nyquist and Bode diagrams. Generally, Nyquist graphs do not show frequency information, and Bode graphs as a complementary way can help us to offer the better understanding of the effect of laser parameters for varied applications. We will discuss the effect of pulse frequency via topography and material characterization techniques to describe how changes in the frequency as one of the most important fabrication parameters, affect the T3DN properties. Additionally, the theoretical model has been customized, and we show that both experimental and theoretical results are in good agreement. The results presented in this work can lead to promising solutions for using laser pulses for the fabrication of better nanostructured titania with predetermined electrochemical properties, which can address our needs in different EES applications.

## 2. Materials and Methods

### 2.1. Electrode Materials and Fabrication

Ti electrodes coated with T3DN were fabricated as reported in our previous work [23]. First, Ti sheets were polished and washed with acetone followed by DI water. The pulse ionization process was conducted with 150 picosecond laser pulses system (IPG Laser Model: YLPP-1-150 V-30) at 600 kHz and 1.2 MHz at a constant wavelength of 1060 nm, a power of 10 kW and a scanning speed of 200 mm/s in a circular pattern. The possible maximum and minimum frequencies of the laser system at 150 picoseconds are 1200 kHz and 600 kHz respectively. Then the electrode disks were cut to 10 mm in diameter (area = 0.785 cm^2^) via water jet cutter station. Three types of samples were used for the tests as follows: control sample (S1), 600 kHz disks (S2), and 1200 kHz disks (S3).

### 2.2. Supercapacitor Assembly

To test the electrochemical performance of the materials, asymmetric cells with lithium metal as counter electrodes were fabricated using a split test cell from the MTI Corporation (Richmond, CA, USA). All assembly was performed under an argon-filled glove box. The prepared 10 mm electrodes were sandwiched with Celgard^®^ 2500 separator with 75 uL of 1 M LiPF_6_ in EC:DEC (1:1, *v*/*v*) from Sigma Aldrich (Oakville, ON, Canada).

### 2.3. Material Characterization

Samples were characterized for surface morphology using FEI Quanta 3D 200/600 scanning electron microscopy (SEM, Hillsboro, OR, USA). The chemical compositions of the laser-irradiated samples were analyzed with a Rigaku Ultima IV X-ray diffractometer (XRD, Austin, TX, USA) using Cu Kα radiation (λ = 0.15418 nm, 40 kV, and 44 mA) system and Raman spectroscopy (Renishaw Raman Imaging Microscope System 2000, Renishaw, Mississauga, ON, Canada).

### 2.4. Electrochemical Characterization

Before electrochemical testing, the assembled supercapacitors were categorized into three groups, Type 1, 2, and 3. Lithium metal was used as the reference and counter electrodes in the split cells. These supercapacitors were assembled in the split cells as described in Section 2.2 and tested with CV, galvanostatic charge/discharge, and EIS [15]. CV, EIS, and charge/discharge testing were carried out using PAR 263A Potentiostat/Galvanostat coupled with a Solartron 1260 frequency response analyzer (Solartron, Forestville, NY, USA). The CV was swept at 20, 200, 300, 400, and 500 mV s^−1^ between 0 and 2.5 V. The frequency range of EIS was carried out from 1 Hz to 100 kHz with 100 mV in open circuit potential.

## 3. Results and Discussion

### 3.1. Surface Morphological and Compositional Analysis

**Figure 1:**
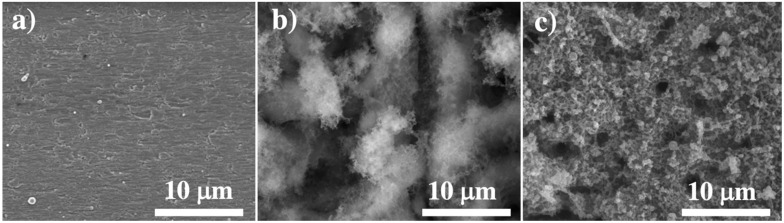
A. SEM images of (**a**) control sample (S1:CS), (**b**) S2 (600 kHz), and (**c**) S3 (1200 kHz).

In SEM images in Figure 1, it can be observed that by changing the laser frequency from 600 to 1200 kHz, the topology of the electrode surface would be varied. Generally, laser-induced porous structures with a very high surface area on the electrode surface can absorb more ions during the charge and discharge process; accordingly, with a higher surface area (higher porosity), better capacitance behaviors can be achieved [23,24,25]. As the frequency increases from 600 to 1200 kHz, the amount of nanofibrous structures and porosity decreases (Figure 1a–c).

The micro topology of samples 2 (S2) and 3 (S3) are shown in Figure 1. We can clearly observe a higher porosity in both micro and nano level in S2; also, we have a slightly larger thickness for samples prepared at 600 kHz (T2) (S2: 69 μm and S3: 43 μm). The porosities of S2 and S3 were measured by ImageJ (v. 1.501 by Wayne Rasband at the National Institutes of Health, Bethesda, MD, USA) to be 59% and 36% respectively with 5% possible errors. It is assumed that the surface porosity can be applied to the volume of active materials which results in density values of 1.85 g/cm^3^ and 2.44 g/cm^3^ for the electrodes processed at 600 and 1200 kHz. Thus, the mass of the active materials for electrodes prepared at 600 and 1200 kHz can be calculated to be 10.02 and 8.24 mg respectively.

**Figure 2:**
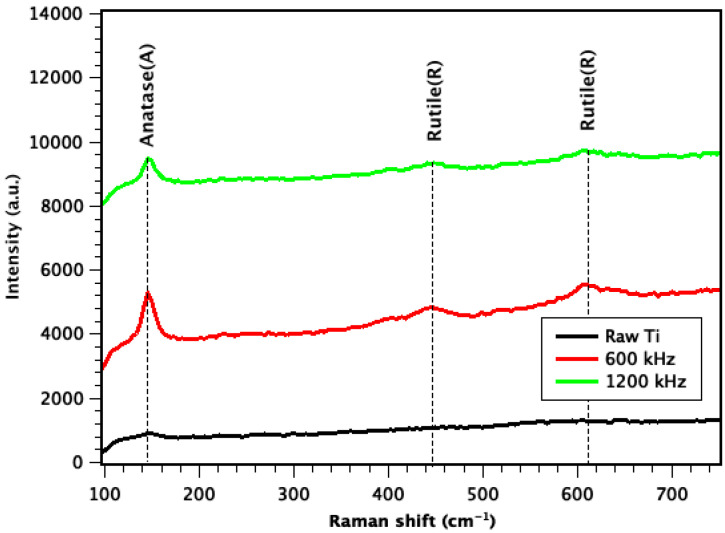
Micro-Raman results of S1, S2, and S3.

The other difference between S2 and S3 is the level of oxidation that occurred during the laser irradiation process, which can be easily observed in micro-Raman results presented in Figure 2. The Raman results in Figure 2, clearly show sharper peaks for both anatase phase at 144 and rutile phase at 443 and 610 cm^−1^ which means a higher presence of Ti oxide in S2 [26].

As reported in our previous research work [23], for the control sample, there are only two main peaks of Ti (101) and Ti (110) observed. For the samples with T3DN, the observed diffraction peaks were indexed to Ti(101), R(111), A(200), R(220), and Ti(110) in accordance to titania anatase (JCPDS card No. 21-1272) and titania rutile (JCPDS card No. 88-1175) [27,28]. It has been already proven in [23] that the peaks of rutile and anatase are sharper in the sample with higher amount of T3DN (S2).

From the SEM, Raman and XRD results, it can be concluded that in S2 there are fewer fibrous structures and higher oxidization rates compared with the Ti sheets processed at 1200 kHz (S3).

The higher oxidation rate at the lower frequency of 600 kHz is because the maximum surface temperature of the irradiated zone at this frequency is higher as each pulse has higher energy. However, at the higher frequency of 1200 kHz, the laser plasma plume is more stable, which results in better agglomeration of the ionized atoms and evaporated nanoparticles to form thicker nanofibrous structures.

### 3.2. Theoretical Models for Surface Temperature

**Figure 3:**
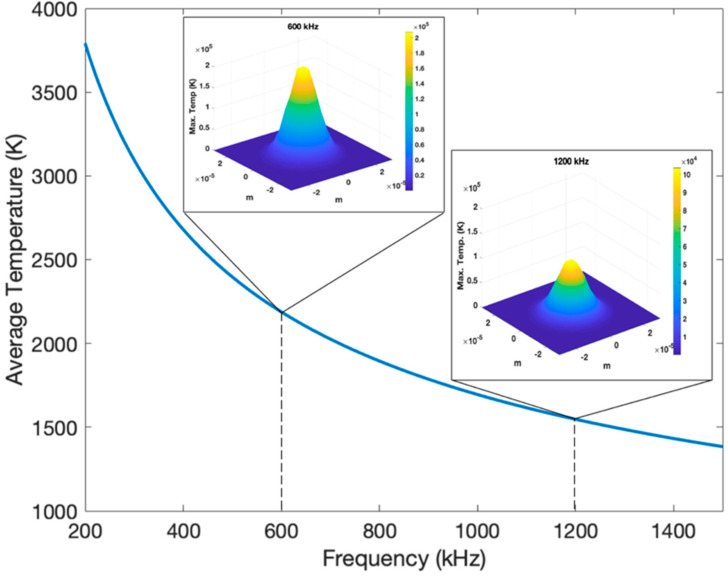
Theoretical results for the average and maximum surface temperature at varied pulse frequencies.

In order to investigate the effect of laser frequency on the plasma plume temperature, a theoretical model [29,30,31] was customized for calculation of the surface temperature of the Ti sheets at varied pulse frequencies (Figure 3).

In our customized theoretical model, the 3D radial temperature gradient is calculated by:

(1) $$\Delta T\left(r,\;z,\;\tau \right)=\frac{I_{max}\gamma \sqrt{\kappa }}{\sqrt{\pi }K}\overset{\tau }{\underset{0}{\int }}\frac{p\left(\tau -t\right)}{\sqrt{t}\left[1+\frac{8\kappa t}{W^{2}}\right]}e^{-\left[\frac{z^{2}}{4\kappa t}+\frac{r^{2}}{4\kappa t+0.5W^{2}}\right]}dt$$

Here, titanium diffusivity is *k*; *r* and *z* are the radius of laser spot and the depth of the laser heat-affected zone respectively, *K* is titanium thermal conductivity *I_max_* is the peak intensity, *γ* is 1-R (R is the Fresnel reflectivity), and *W* is the beam’s (1/e) field radius. Also, *p*(*t*) is assumed to be equal to 1 for square-shaped pulses at the center of ablation.

As we can see here by increasing the frequency of the laser pulses, both the average and maximum surface temperature will be decreased. The theoretical results agree with experimental results as at higher temperature, we expect more oxidation rate in S2 processed at 600 kHz compared with S3 at 1200 kHz. In fact, the frequency as a determinative laser parameter, affects both the surface area (porosity and nanofiber generation) and oxidation levels. In the following section, it will be explained how these two factors influence the electrochemical properties of supercapacitors.

### 3.3. Supercapacitor Application

#### 3.3.1. Cyclic Voltammetry

The cyclic voltammetry technique, which can provide a vast amount of information in a short period, was used to evaluate the electrochemical properties in the different applications such as energy storage and supercapacitor fabrication.

**Figure 4:**
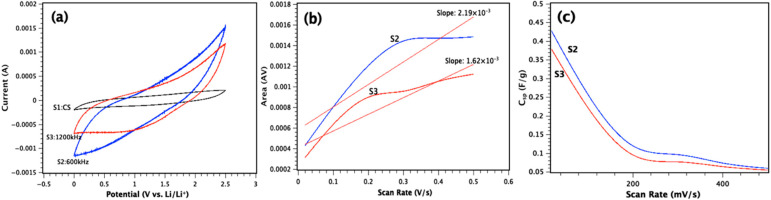
(**a**) CV plots of three types of electrodes (S1, S2, and S3) at 500 mV/s; (**b**) Are (AV) versus scan rate (V/s); (**c**) C_sp_ (F/g) versus scan rate (mV/s).

The CV curves of T3DN electrodes in 1M LiPF_6_ in EC:DEC have a nearly rectangular shape from scan rates 200 mV/s to 500 mV/s, indicating appropriate capacitive behavior (Figure 4a). Electrochemical performance also depends on the nature of the electrolyte. However, we did not focus on this aspect as it was identical for all tests. A general improvement can be seen in S2 compared to S3 as shown by the results presented in Figure 4. We concluded that this improvement is from the laser frequency effect on both topology and oxidation levels. Lower laser frequency can produce a higher thickness in electrode fabrication based on a comparison between CV curves of the supercapacitors. As we can see in Figure 4, the integrated area for S2 is significantly larger than areas for T3 and T1 calculated by OriginPro in CV graphs. The specific capacitance of the prepared supercapacitors can be calculated as follows [32]:

(2) $$C_{sp}=\frac{A}{2\;\left(V2-V1\right)\left(\frac{\Delta V}{\Delta t}\right)m}$$

where, *A* is the area under (AV), $\frac{\Delta V}{\Delta t}$ is the scan rate (V/s), m is the weight of electrode and *V*1 and *V*2 (V) are the applied voltages in the tests. Thus, specific capacitances of S2 and S3 at 500 mV/s are calculated to be 59.85 and 54.39 mF/g. The parameters for other scan rates can be found in Table 1. Alternatively, *C_sp_* for S2 and S3 can be calculated as follows:

(3) $$S=2\left(V_{2}-V_{1}\right)\times m\times C_{sp}$$

where, *S* is the slope of the fitting line in the “area vs. scan rate” as shown in Figure 4b. Thus, the *C_sp_* values for S2 and S3 are calculated to be 43.6 and 39.2 mF/g, respectively.

The results for *C_sp_* at different scan rates, from 20 to 500 mV/s, have been presented in Figure 4c. The results show that the specific capacitance decreases by increasing the scan rate from 20 to 500 mV/s which can be due to different factors such as pore-size distribution, the presence both of micro- and mesopores, and specific surface area as reported before [33,34,35,36]. In both S2 and S3, charged nanofibrous structures have more time at lower scan rates to interact with other fibers and pores of T3DN electrodes. It is clear in the CV results that the higher amount of T3DN and higher oxidation rate in S2 improves the *C_sp_* retention that favors the higher power density as presented in Figure 4c and the highest value for *C_sp_* is observed for S2 to be 427.5 mF/g at 20 mV/s. In fact, the higher generation of T3DN helps in better electron transportation during the charge-discharge process. This depicts a better capacitance for the S2 electrode prepared at the lower frequency of 600 kHz, showing that it is possible to customize the capacitive behavior of T3DN electrodes by varying laser parameters (pulse frequencies) that directly affect the 3D-Nanonetwork fabrication and oxidation levels in the generated active materials on electrode surfaces.

#### 3.3.2. Charge/Discharge

**Figure 5:**
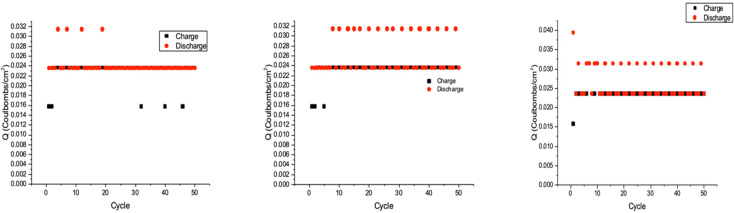
Coulombs versus number of cycles in charge/discharge process.

As we can see from charge/discharge, which is depicted in Figure 5, the number of jumps (to Q = 0.032 C/cm^2^) in three samples. As mentioned in the experimental setup, we used two individual electrodes for all three supercapacitor types, which means we may have similar but slightly different behaviors on two sides (electrodes in each setup). If we assign the names *C_negative_* and *C_positive_* to the specific capacitance of negative and positive electrodes, we can consider this equation:

(4) $$C_{abs}=\left|C_{negative}-C_{positive}\right|$$

*C_abs_* is the absolute difference of electrode capacitance. By comparing three charge/discharge graphs, we concluded that an increase in *C_abs_* leads to a higher number of jumps. Jump numbers here correspond to the saturation time of the electrode and can be used as qualitive data to the energy storage devices’ behaviors [33]. For instance, in S2 and S3, we have 16 and 21 jumps in the first 50 cycles, respectively; the jump number is only 4 in S1 (control sample) through the capacity saturation of electrodes. The S1 supercapacitor needs less time to become saturated during the charge and discharge cycles and has the lowest capacitance compared with S2 and S3 [34]. Considering both electrode capacitance and saturation time, S2 performs better compared with the other two electrodes (S1 and S3)

(5) $$(C_{positive,I}=n_{S1}\times C_{negative,I},\;C_{positive,\mathrm{II}}=\;n_{S2}\times C_{negative,\mathrm{II}},\;C_{positive,\mathrm{III}}=n_{S3}\times C_{negative,\mathrm{III}})\;\to \;n_{S3}>n_{S2}>n_{S1}$$

#### 3.3.3. Electrochemical Impedance Spectroscopy

**Figure 6:**
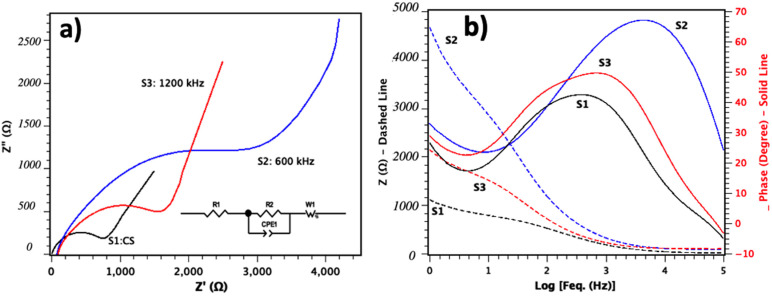
(**a**) Nyquist plots (black: CS, Blue: S2 and Red: S3) and (**b**) Bode plots of S1, S2 and S3 (Z: dashed line, Phase: solid line).

Electrochemical impedance spectroscopy measurements were conducted for the bare titanium (S1:Control Sample) and T3DN electrodes (S2 and S3) at a frequency range from 1 Hz to 100 kHz and its Nyquist plot is shown in Figure 6a. From the results, it can be observed that all three samples showed a semicircle in the high-frequency region. For S2 we observed a higher radius for the semicircle regions, which can be attributed to the resistance to electron transfer within the electrode materials as a result of higher oxidation levels in electrodes (S2) fabricated at pulse frequency of 600 kHz. The total impedance of the supercapacitor depends on both electronic and ionic contributions [37,38]. The electronic contribution is controlled by the intrinsic resistance to electron transfer and interfacial resistances of the particles. The ionic contribution is related to the diffusion resistance of ions toward pores as well as the electrolyte resistance in the pores [38,39,40]. Compared to S1 and S3, the low frequency region of S2 exhibits a vertical line, which is due to the ion’s better diffusion in the electrolyte to the electrode interface, resulting in the better performance of the T3DN electrodes fabricated at 600 kHz (S2).

As shown in the Bode plot in Figure 6b, the total impedance decreases with increasing frequency at low frequency regions. On the other hand, the frequency independent impedance region is observed at higher frequency which is related to the diffusion resistance of electrolyte ions into the electrodes [40,41,42]. Generally, at high frequencies, the S2 and S3 electrodes have higher capacitance behavior compared to the control sample (S1).

Also, in the Bode plot, the highest phase angle of S2 is −67° which is closer to the phase angle of ideal capacitor (−90°) in comparison with S3 (−48°) and S1 (−41°). The capacitor response frequency (f_0_) could be acquired at a phase angle of −45°, from which the relaxation time constant (*τ*_0_ = 1/f_0_) can be calculated to be 0.25 ms and 1.56 ms for T2 and T3 respectively.

According to equivalent circuit model employed in this study in Figure 6a, a serial connected R1, R2||CPE2 and W1 was created. In this model, the first compound, R1 belongs to the Ti sheet (substrate), and R2||CP2 corresponds to the T3DN layer. According to previous studies [43,44], constant phase element (CPE) is a parameter which can be used to determine if the circuit element is behaving between capacitor and resistor. CPE involves pseudo capacitance which is called Q or CPE-T and CPE-P, a factor related to the depressed semicircle in the Nyquist plot. CPE-T corresponds to the amplitude and the values of CPE-P vary in the range of 0 to 1 (zero for the ideal resistor and 1 for the ideal capacitor). In summary, in the supercapacitor, lower R2 and CPE-T and higher value closer to 1 for CPE-P are desirable.

**Figure 7:**
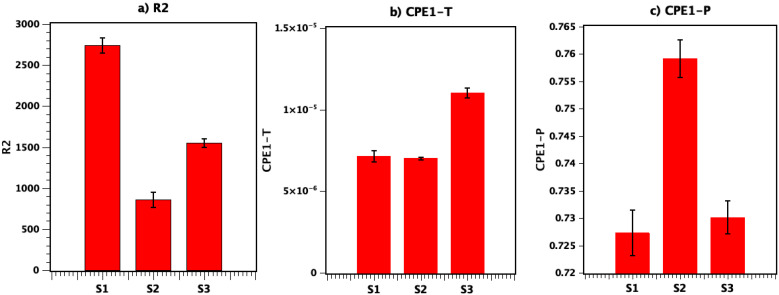
R2, CPE-T and CPE-P for S1, S2, and S3 (error bar marked on the graph representing standard deviation).

As shown in Figure 7, for S2, CPE-P has the closer value (0.76) to 1 and CPE-T has a minimum value compared to the S3 and S1.

In Figure 7, the results also confirm the lowest resistivity (R2) for S2 in comparison with the other samples. In summary, S2 has the best results for CPE-T, CPE-P, and R2 in comparison with S3 and S1 (which has the lowest performance); the indicated changes are due to the higher amount of generated T3DN in S2 as well as its higher oxidation level. As previously mentioned, at the frequency of 600 kHz the temperature of the fabrication process is higher, which results in generation of a thicker layer of T3DN with higher porosity on electrode sheets. In general, a higher surface area and more oxidized Ti results in a higher surface area and better ion diffusion in electrodes. In the proposed method, both the topology and oxidation levels of the generated T3DN can be controlled by fabrication parameters including frequency, pulse duration, intensity, and number of pulses. In this paper, we mainly focus on the effect of the frequency and in the future direction of this research, other parameters for the fabrication of optimized advanced electrodes will be investigated.

## 4. Conclusions

In this study, we investigated the effect of pulse frequency as a key fabrication parameter on the electrochemical performance of T3DN electrodes. We varied the pulse frequency from 600 to 1200 kHz and conducted both morphology and material characterization tests to evaluate the morphology of the generated nanostructures and their compositions. We found that by decreasing the pulse frequency, more nanofibrous structures can form with higher oxidation rates. This observation was proven through the theoretical models for calculation of average and maximum surface temperature.

The higher surface area and oxidation rate in electrodes prepared at 600 kHz results in better electrochemical performances, and this has been carefully studied using CV, charge/discharge, and EIS analysis. The specific capacitances of samples fabricated at 600 kHz and 1200 kHz were calculated to be 59.85 and 54.39 mF/g at 500 mV/s, respectively. We found that by controlling the pulse frequency, the performance of the supercapacitors using the T3DNs could be customized for different applications. We strongly believe this pulse ionization method can open up new doors to this emerging and fast-growing field by introducing new techniques for EES and supercapacitor fabrication. Our proposed method is single step and environmentally friendly as no chemicals are used in the fabrication process and it can be done under ambient conditions. Also, the T3DN can grow directly on the Ti sheets without the use of any binder or conducting agent active material, which reduces the fabrication time and cost and can significantly minimize the resistance between the current collector (Ti) and active materials. In the future direction of this study, the other laser parameters (e.g., pulse duration, pulse power, pulse number) will be investigated to determine the optimized parameters for fabrication of advanced supercapacitor electrodes with predetermined electrochemical properties.

## Figures and Tables

**Table 1:** *C_sp_* and area in CV plots for S2 and S3 at different scan rates.

Sample	Scan Rate (V/s)	Potential Window (V)	Mass of Active Material (g)	Area (AV)	*C_sp_* (F/g)
S2	0.500	2.5	0.01002	0.0014826	0.05918563
	0.400			0.001469	0.07330339
	0.300			0.001442	0.09594145
	0.200			0.0012018	0.11994012
	0.020			0.0004284	0.42754491
S3	0.500	2.5	0.00824	0.001120548	0.05439553
	0.400			0.001050816	0.06376311
	0.300			0.000954892	0.07644757
	0.200			0.00089702	0.09551214
	0.020			0.000312004	0.37864563

## Data Availability

The data presented in this study are available on request from the corresponding author.
